# The relationship between risky sexual behaviors and sexual health literacy and self-esteem in young women

**DOI:** 10.1590/1806-9282.20241040

**Published:** 2025-03-31

**Authors:** Sevil Cicek Ozdemir, Esra Cevik

**Affiliations:** 1 Kütahya Health Sciences University, Faculty of Health Sciences, Department of Nursing – Kütahya, Turkey.; 2 Balikesir University, Faculty of Health Sciences, Department of Midwifery – Balıkesir, Turkey.

**Keywords:** Respect, Health literacy, Sexual behavior, Women

## Abstract

**OBJECTIVE:**

The aim of this research was to examine the relationship between risky sexual behaviors and sexual health literacy and self-esteem in young women.

**METHODS:**

The research is descriptive and cross-sectional type. In total, 705 young women were included in the research. The study data were collected online using an online survey system through Google Forms. The data were collected between April and July 2023.

**RESULTS:**

The factors affecting premarital risky sexual behavior were determined to be the educational level of the woman and her mother, where the women lived the longest, current residence, family status, father's educational level, employment status of the woman and her mother, and smoking and alcohol use status. Using alcohol (13 times), smoking (4 times), and mother's employment status (3 times) have the highest effect on the mean Premarital Sexual Behavior Assessment Scale for Young Women score. There is a significant negative correlation between Premarital Sexual Behavior Assessment Scale for Young Women and Sexual Health Literacy Scale. There is no significant relationship between Premarital Sexual Behavior Assessment Scale for Young Women and Rosenberg Self-Esteem Scale.

**CONCLUSION:**

Increasing sexual health literacy levels, quitting smoking and alcohol, and engaging in self-esteem-enhancing activities in women are essential for reducing risky sexual behaviors.

## INTRODUCTION

Sexuality is one of the basic human needs. Sexuality is a normal, healthy, and integral part of human life. Among young adults, sexuality is an important aspect of their lives^1^. Young people are vulnerable to risky sexual behaviors due to physical, cognitive, sexual, behavioral, emotional, and social developments^2^.

In developing countries with changing social, cultural, and moral norms, such as Turkey, the taboo of premarital sexuality and the associated sexual drive and curiosity can lead young people to risky sexual behaviors^3^. Risky sexual behavior is generally defined as increasing the risk of contracting sexually transmitted infections and having unwanted pregnancies^4^. These behaviors include early sexual intercourse, unprotected vaginal, oral, or anal intercourse, multiple sexual partners, sexual intercourse for money or under the influence of substances, and unsafe contraceptive method use (irregular condom use)^5^.

Many factors affecting risky sexual behavior are mentioned in the literature. Srahbzu and Tirfeneh stated that socioeconomic status, unemployment, having sexually active friends, relationships with family, single-parent home, and sibling sexual activity are among the reasons why adolescents are involved in risky sexual behaviors^6^. Shrestha stated that the factors contributing to risky sexual behaviors include parental relationship, cultural and traditional rules and values, school environment, peer pressure, and social media^7^. It is also stated that gender, depression, and anxiety cause risky sexual behaviors^8^.

Sexual health literacy and self-esteem are other factors affecting risky sexual behaviors^9^. Sexual health literacy is crucial in maintaining sexual health and preventing sexually transmitted diseases. Women need information to ensure the continuation of their sexual life quality, to recognize sexual health problems, and for early diagnosis and treatment of sexual health problems^10^. In the studies conducted in the literature, there are studies reporting that individuals with high sexual health literacy tend to have less risky sexual behavior^11,12^. However, there are also studies reporting that those who receive information about sexual health tend to have more risky sexual behavior^13^ or that there is no relationship between sexual health literacy and risky sexual behavior^14^. These different results in the literature reveal that the relationship between risky sexual behaviors and sexual health literacy is not clear, and this situation needs to be clarified.

In the literature, self-esteem has also been associated with risky sexual behaviors such as unsafe sex practices, multiple sexual partners, same-sex intercourse, and rape^15^. Self-esteem is the individual's thoughts and evaluations about their life and experiences, self-love, and self-respect by being aware of their positive and negative characteristics^16^.

Although men are more prone to risky sexual behaviors^17^, the physical, psychological, and social consequences on women make women's lives even more risky. Risky sexual behaviors cause physical effects such as unwanted pregnancies, abortion, induced and unhealthy miscarriage, sale of babies, sexually transmitted diseases, and maternal death, as well as psychological and social consequences such as depression, suicide attempts, and dropping out of school^3^. These behaviors can also directly affect the set of educational and cultural assets that determine an individual's well-being. Therefore, a better understanding of the factors of risky behavior during adolescence is important for interventions to prevent this pattern of behavior in adulthood. Those who engage in any risky behavior are likely to engage in other risky behaviors, and there may be common factors that predict the development of these behaviors. For this reason, a growing body of research shows that risk behaviors often do not occur in isolation. Smoking, drinking, illicit drug use, sexual risk-taking, and aggression are mutually predictive behaviors^18^. Therefore, risky sexual behaviors are a significant concern for educators, psychologists, and researchers^3^. This study examines the relationship between risky sexual behaviors, sexual health literacy, and self-esteem in young women.

## METHODS

### Types of research

The research is descriptive and cross-sectional type.

### Population and sample of the research

The research population consists of women aged 18–34 years living in Turkey. When the universe of the research was taken as 9,580,733,601 was calculated for the study sample with a prevalence of 50%, a margin of error of 4%, and a confidence interval of 95%. In total, 705 young women were reached. Women between the ages of 18 and 34 years, literate, single, able to use the internet, and who agreed to participate were included in the study.

### Place and time of the research

The study data were collected online using an online survey system through Google Forms. The data were collected between April and July 2023.

### Data collection form

The questions in the data collection form were converted into an online survey form.

Descriptive Information Form: The form includes 18 questions.

Premarital Sexual Behavior Assessment Scale for Young Women (PSAS-YW): The Turkish adaptation of the scale developed by Rahmani et al. in Iran^19^ was conducted by Kaydirak et al.^20^. The Likert-type scale consists of 20 items and is scored as "1" for strongly agree and "5" for strongly disagree. The minimum and maximum score is 20–100. There is no cut-off score or degree of risky sexual behavior for the Turkish version. The risk decreases as the score obtained from the scale increases.

Sexual Health Literacy Scale (SHLS): The scale developed by Üstrgörül consists of 17 items. The scale uses a 5-point Likert-type scale ranging from Strongly Disagree (1) to Strongly Agree (5). The scale has a two-dimensional structure for sexual knowledge and sexual attitude. The lowest score and highest are 12–60. An increase in the scores obtained from the subdimensions of the scale and the overall scale indicates high sexual health literacy^21^.

Rosenberg Self-Esteem Scale (RSES): This scale was developed by Rosenberg^22^. The scale consists of 12 subcategories and a total of 63 questions structured from multiple-choice questions, and the first "10" items are used to measure self-esteem. There are response options for the scale items as "1: Very true", "2: True", "3: False" and "4: Very false." If the total score obtained from 10 items is 0–1, self-esteem is evaluated as high, 2–4 as moderate, and 5–6 as low.

### Data collection

The study was conducted online since our research group had both insufficient health-seeking behaviors (applying to a health institution) and high rates of internet use. The developed scale form was converted to an online questionnaire via Google Forms^®^. The study was carried out by sending online links to nurses through social networking sites such as Facebook^®^, WhatsApp^®^, and Instagram^®^. Besides, researchers shared the link on forums and blogs on the Internet.

### Data assessment

The data obtained in the study were transferred to the SPSS 23.0 package data program and evaluated. Descriptive statistics such as number, percentage, mean, standard deviation, t-test in independent groups, one-way ANOVA, and post hoc analysis (post hoc: Tukey HSD) were used to analyze the data. Pearson correlation analysis and multiple regression analysis were also applied.

### Ethical aspects of the research

Ethics committee permission was obtained from Balikesir University University Clinical Research Ethics Committee to implement the study (date: 27.04.2023; number: E-11811414-050.03-251150). Participants could participate in the study after reading the informed consent form included in the data collection form and giving their consent.

## RESULTS

The mean PSAS-YW score of young women with bachelor's and postgraduate education was significantly lower than those with high school and associate degree education (F=4.95; 0.001). Women who lived in the villages/towns for the longest (F=12.79; 0.000) and lived with their families (F=9.78; 0.000) had significantly higher mean scores. The mean score of women whose parents were alive but separated and whose mother was alive but whose father was not alive was significantly lower (F=7.63; 0.000). Women whose mothers were college or university graduates had substantially lower mean scores than those whose mothers had other levels of education (F=7.96; 0.000). The mean score of women who were employed (t=-2.97; 0.003), whose mothers were employed (t=0.62; 0.000), smoked (t=-7.95; 0.000), and used alcohol (t=-17.25; 0.000) was significantly lower (Table 1).

**Table 1:** Some characteristics affecting the mean Premarital Sexual Behavior Assessment Scale for Young Women score of young women.

Characteristics		Mean±SD	Statistics/p-value p
Educational status			
	Primary and secondary education	78±23.19	F=4.95 **0.001** a,b>c,d
	High school graduate^a^	72.07±14.57	
	Associate degree^b^	75.18±15.19	
	Bachelor's degree^c^	68.82±13.88	
	Postgraduate^d^	67.22±14.92	
Employment status			
	Yes	68.39±14.01	t=-2.97 **0.003**
	No	71.82±14.99	
Where the women lived the longest			
	Province^a^	68.69±14.53	F=12.79 **0.000** c>a,b
	District^b^	73.27±14.29	
	Village or town^c^	76.29±14.69	
Current residence			
	Living alone^a^	64.42±13.87	F=9.78 **0.000** c>a,b,d
	Living with friends^b^	66.18±15.29	
	Living with family^c^	72.52±14.32	
	Living with partner^d^	64.11±16.27	
Mother's employment status			
	Yes	65.74±14.34	t=0.62 **0.000**
	No	72.56±14.44	
Family structure			
	Both are alive, living together^a^	71.27±14.5	F=7.63 **0.000** d> a>b,c
	Both alive, living separately^b^	63.41±14.24	
	Neither of them are alive	70±9	
	My mother is alive, my father is not^c^	64.11±14.24	
	My father is alive, my mother is not^d^	84.81±13.81	
Mother's educational status			
	Illiterate^a^	74.14±14.02	F=7.96 **0.000** a, b>d,e c,d>e
	Literate or primary education^b^	72.76±15	
	Secondary education^c^	69.99±14.08	
	High school graduate^d^	68.32±14.19	
	College or university^e^	62.85±12.84	
Father's educational status			
	Illiterate	73.22±13.7	F=2.80 **0.025**
	Literate or primary education	72.43±15.1	
	Secondary education	71.89±14.29	
	High school graduate	70.01±14.33	
	College or university	67.56±14.79	
Smoking status			
	Yes	62.06±14.82	t=-7.95 **0.000** **0.000**
	No	72.69±13.94	
Alcohol consumption status			
	Yes	56.64±9.87	t=-17.25 **0.000**
	No	74.09±13.64	

SD: standard deviation. Statistically significant values are indicated in bold. Letters (a–e) indicate differences between groups.

There is a significant negative correlation between PSAS-YW and SHLS (r=-0.09; p=0.009). There is no significant relationship between PSAS-YW and RSES (r=-0.07; p=0.052) (Table 2).

**Table 2:** The relationship between Premarital Sexual Behavior Assessment Scale for Young Women, Sexual Health Literacy Scale, and Rosenberg Self-Esteem Scale scales of young women.

		PSAS-YW	SHLS	RSES
PSAS-YW	**r**	1	-0.09	-0.07
	p		**0.009**	0.052
SHLS	r	-0.09	1	-0.08
	p	**0.009**		**0.021**
RSES	r	-0.07	-0.08	1
	p	0.052	**0.021**	

PSAS-YW: Premarital Sexual Behavior Assessment Scale for Young Women. SHLS: Sexual Health Literacy Scale. RSES: Rosenberg Self-Esteem Scale. Statistically significant values are indicated in bold.

According to the multiple regression analysis with the factors affecting the mean PSAS-YW score, the model was statistically significant (F=28.85; p=0.000), and the variables explained 26.3% of the model. Lived the longest (ß=-1.80; p=0.014), current residence (ß=1.82; p=0.006), mother's employment status (ß=3.08; p=0.007), smoking (ß=4.17; p=0.002), and using alcohol (ß=13.85; p=0.000) affect the mean PSAS-YW score. Using alcohol (13 times), smoking (4 times), and mother's employment status (3 times) have the highest effect on the mean PSAS-YW score (Table 3).

**Table 3:** Regression analysis of factors affecting Premarital Sexual Behavior Assessment Scale for Young Women mean scores.

Model	ß	Std. error	Beta	t	p-value	Tolerance	VIF
Constant	39.75	5.31		7.48	0.000		
Educational status	-0.76	0.48	-0.06	-1.57	0.117	0.63	1.57
Employment status	-1.96	1.30	-0.06	-1.50	0.132	0.58	1.71
Where the women lived the longest	-1.80	0.73	-0.08	-2.47	**0.014**	0.90	1.10
Current residence	1.82	0.66	0.09	2.74	**0.006**	0.81	1.22
Mother's employment status	3.08	1.13	0.09	2.72	**0.007**	0.86	1.15
Family structure	-0.05	0.57	-0.00	-0.09	0.927	0.96	1.03
Mother's educational status	-0.53	0.47	-0.04	-1.12	0.261	0.81	1.23
Smoking	4.17	1.32	0.11	3.15	**0.002**	0.82	1.21
Using alcohol	13.85	1.37	0.37	10.08	**0.000**	0.75	1.33

F=28.85; p=0.000; Adjusted R^2^=0.26; Durbin-Watson katsayısı=1.89. Statistically significant values are indicated in bold. VIF: variance inflation factor.

## DISCUSSION

Examining sexual expression in different cultural settings sheds much light on the similarities and differences of the same phenomenon across cultures^23^. In Turkey, there is still social pressure on women to have premarital sex, sexuality is perceived as taboo, forbidden/shameful, and sexual issues cannot be discussed openly, which results in limited information about the sexual behavior profile. Despite these situations, the age of first sexual intercourse is decreasing in Turkey, and high-risk sexual behaviors such as premarital/extramarital sex and sex work, multiple sexual partners, sex without a condom, and early sexual activity are important areas of research^24,25^. Some health programs designed to prevent and treat specific risk behaviors in young people suggest that these behaviurs are shaped by four socialization units: family, school, religious and community institutions, and peers. There is also evidence that individual personality traits and temperament are determinants of risky sexual behaviors^18^. Understanding the risk factors associated with risky sexual behaviors can lead to effective and timely identification and intervention for those at greatest risk.

Our study found that young women living with their families had a lower risk of premarital risky sexual behavior. In a survey conducted on 599 individuals between the ages of 15 and 24 years in Nigeria examining the effect of family support and living with both parents on sexual behaviors, it was reported that living with both parents was positively associated with protective sexual behaviors^26^. Similarly, a study found that living with both parents had a protective effect regarding risky sexual behaviors^27^. The results of this study are in parallel with the results of our research. Again, our study found that young women whose parents were alive but separated and whose mother was alive but whose father was not alive had a higher risk of premarital risky sexual behavior. Similarly, the study identified that being a single parent was a reason for risky sexual behaviors^6^. In the literature, various family-related factors such as harsh parenting, low parental control, and family cohesion have been identified as risk factors for sexual risk-taking behavior. Low parental control, low impulsive control, early maltreatment, negative emotions, and risky behavior occur in the following order^28^. The literature states that parental relationships, cultural and traditional rules, and values are effective in risky sexual behaviors^7^. However, unlike our research result, in a study conducted with 6,167 high school students aged 13–19 years in Thailand, no significant difference was found between parental divorce and risky sexual behaviors such as sexual intercourse^29^. This may be interpreted as a result of the multidimensional nature of sexuality and sexual behaviors.

In support of our study, Okeke et al. found that family, parental, economic, religious, and social factors were associated with risky sexual and health behaviors of young people^3^. In another study, socioeconomic status and unemployment were cited as the reasons for adolescents’ involvement in risky sexual behaviors^6^. In a review study conducted in the literature, it was stated that educational status, economic status, and place of residence characteristics affect sexual and reproductive health outcomes^30^.

Our study found that young women who smoked and used alcohol had a higher risk of premarital risky sexual behavior. In some studies in the literature, it has been emphasized that young people who use alcohol and drugs are more prone to risky sexual behaviors^6,31^. These studies’ results support our research results. In addition, our study determined that alcohol (13 times) and smoking (4 times) increased the risk of premarital sexual behavior. It is thought that the higher risk of experiencing sexual intercourse and engaging in risky sexual behaviors in young people who smoke and use alcohol is related to being under the influence of these substances. The results of a study conducted with 33,744 college students in the literature confirm this. According to this study, compared to students who do not smoke or consume alcohol, smoke 1–9 cigarettes a day, and consume 1–6 glasses of alcohol per day, it was determined that the group "smoking more than 10 cigarettes and consuming more than 7 glasses of alcohol per day" had a higher risk of having sexual intercourse^32^. Similar to our research results, unlike other protective factors (familial attachment, social support, self-esteem), drug and alcohol use has a direct and strong positive effect on sexual risk taking, and young people who report higher levels of drug and alcohol use also tend to take higher levels of sexual risk^33^. Those who engage in any risky behavior are likely to engage in other risky behaviors, and there may be common factors that predict the development of these behaviors. Smoking, drinking, illicit drug use, sexual risk-taking and aggression are mutually predictive behaviors^18^.

Exposure to violence may pave the way for health risks such as risky sexual behavior and substance use. In the research group, it was found that there was no relationship between the risk of premarital sexual behavior and exposure to violence. In a study conducted by Smith et al. on 127,513 adolescents aged 12–15 years in a multi-country study, it was reported that those exposed to violence had a higher rate of risky sexual behavior^34^. Although the reasons for the differences between countries in the prevalence of violence and risky sexual behavior are unknown, there was no significant difference in our study findings, which may be related to cultural and religious factors. In addition, the fact that our study group had a very low exposure rate to violence in the last month (3.4%) may have affected this situation.

One factor that influences behavior is cognitive factors; adolescents who have accurate and proportionate experience with reproductive health tend to understand behavioral risks and alternative ways to handle them correctly^35^. Similar to our research result, the literature reports that individuals with high sexual health literacy tend to have less risky sexual behavior^36^. Poorer health literacy is associated with poorer health knowledge, worse health outcomes, inadequate use of health services, and increased health care costs^37^. However, studies also report that those who receive information about sexual health tend to have more risky sexual behavior^13^ or that there is no relationship between sexual health information and risky sexual behavior^14^. This difference in the literature is due to the characteristics of the studied group and society. In addition, it is thought that the time, scope, content, and from whom the information is received are important in this difference. In a systematic review of quantitative studies on university students by Arsandaux et al., there were positive associations between self-esteem and having several sexual partners. In terms of sexual behavior, there were negative associations between self-esteem and risky sexual behavior^38^. In the study in Enugu state, it was emphasized that risky sexual and health behaviors were associated with low self-esteem^3^. In a study examining the relationship between risky sexual behavior and self-esteem in university students in Uganda, it was concluded that university students with high self-esteem had a low likelihood and tendency to engage in risky sexual behaviors^39^. Our study found that the risk of premarital risky sexual behavior was not affected by self-esteem. Considering that self-esteem is more likely to be negatively affected during adolescence, this difference is thought to be due to the high average age of our research group. In addition, it is reported in the literature that self-esteem is linked to risky sexual behaviors by reducing alcohol and substance use, rather than directly affecting risky sexual behaviors^33^.

## CONCLUSION and RECOMMENDATIONS

Risky sexual behaviors such as early sexual debut, unsafe sex (e.g., inconsistent condom use), and multiple sexual partners (e.g., high partner switching rate) have attracted worldwide attention due to their long-term adverse health effects, especially sexually transmitted infections, other diseases, unintended/teenage pregnancy, and substance use. Therefore, there is a need to understand the antecedents of risky sexual behaviors for early prevention, which is one of the best strategies to combat later negative outcomes^28^. There is a relationship between premarital risky sexual behavior and the educational level of the woman and her mother, where the women lived the longest, current residence, family status, father's educational level, employment status of the woman and her mother, and smoking and alcohol use status. While there was a significant negative correlation between premarital risky sexual behavior and sexual health literacy, there was no significant correlation between self-esteem. Increasing sexual health literacy levels, quitting smoking and alcohol, and engaging in self-esteem-enhancing activities in women are essential for reducing risky sexual behaviors.

In the literature, there are some interventions such as Masters and Johnson's sensate focus, mindfulness, and cognitive–behavior therapy that focus on changing sexual behaviors and are used to improve sexual health^40,41^. These interventions are important as a better understanding of the factors of risky behavior during adolescence is aimed at preventing this pattern of behavior in adulthood. Additionally, public awareness of risky sexual behavior should be increased, particularly among late adolescents and young adults, but much earlier in life should also be targeted.

### Limitations and strengths

The results of this study are limited to women with internet access. Therefore, it is important to investigate risky sexual behaviors in young women who do not have internet access or who are not attending school. In addition, the factors affecting risky sexual behaviors in this study are limited to socio-demographic characteristics, violence, smoking, and alcohol use. Despite these limitations, this study provides useful evidence to guide future research on preventing or reducing risky sexual behaviors among young women in Turkey. It is also strong in terms of contributing to the conflicting research results on sexual health literacy and self-esteem, which are thought to affect risky sexual behavior and are becoming a global health problem.
